# Elementary and macroscopic light-induced currents and their Ca^2+^-dependence in the photoreceptors of *Periplaneta americana*

**DOI:** 10.3389/fphys.2014.00153

**Published:** 2014-04-22

**Authors:** Esa-Ville Immonen, Stephan Krause, Yani Krause, Roman Frolov, Mikko T. Vähäsöyrinki, Matti Weckström

**Affiliations:** Division of Biophysics, Department of Physics, University of OuluOulu, Finland

**Keywords:** *Periplaneta*, cockroach, photoreceptor, phototransduction, quantum bump

## Abstract

In a microvillar photoreceptor, absorption of an incident photon initiates a phototransduction reaction that generates a depolarizing light-induced current (LIC) in the microvillus. Although in-depth knowledge about these processes in photoreceptors of the fruitfly *Drosophila* is available, not much is known about their nature in other insect species. Here, we present description of some basic properties of both elementary and macroscopic LICs and their Ca^2+^-dependence in the photoreceptors of a dark-active species, the cockroach *Periplaneta americana*. Cockroach photoreceptors respond to single photon absorptions by generating quantum bumps with about 5-fold larger amplitudes than in *Drosophila*. At the macroscopic current level, cockroach photoreceptors responded to light with variable sensitivity and current waveform. This variability could be partially attributed to differences in whole-cell capacitance. Transient LICs, both elementary and macroscopic, showed only moderate dependence on extracellular Ca^2+^. However, with long light pulses, response inactivation was largely abolished and the overall size of LICs increased when extracellular Ca^2+^ was omitted. Finally, by determining relative ionic permeabilities from reversals of LICs, we demonstrate that when compared to *Drosophila*, cockroach light-gated channels are only moderately Ca^2+^-selective.

## Introduction

Visual phototransduction is a G-protein-mediated biochemical cascade that converts the energy of an incident photon to an electrical signal in the plasma membrane of a photoreceptor cell (Fain et al., 2010). In microvillar photoreceptors of arthropods, production of this signal leads to membrane depolarization (Hardie and Postma, 2009). At first, studying the mechanisms underlying this depolarization relied heavily on photoreceptors in the *Limulus* ventral eye (Dorlöchter and Stieve, 1997; Lisman et al., 2002). Since then the development and progress in electrophysiological (Hardie, 1991) and molecular biological methods has enabled studies in the fruitfly *Drosophila melanogaster*, which has become the model system of microvillar phototransduction (Hardie and Raghu, 2001).

In *Drosophila* photoreceptors, phototransduction is triggered when visual pigment rhodopsin, located in the microvillar membrane, absorbs a photon and undergoes a photoisomerization to metarhodopsin. Metarhodopsin activates a heterotrimeric G-protein (G_q_), which, in turn, activates the effector enzyme phospholipase C (PLC). The active PLC then hydrolyses a membrane lipid component phosphatidylinositol 4,5-bisphosphate (PIP_2_) into inositol 1,4,5-trisphosphate (IP_3_) and diacylglycerol (DAG). Transient receptor potential (TRP) and TRP-like (TRPL) channels are then opened apparently by both chemical (Chyb et al., 1999; Huang et al., 2010) and mechanical means (Hardie and Franze, 2012), resulting in depolarizing influx of Ca^2+^ and Na^+^ that generate the light-induced current (LIC) (Niemeyer et al., 1996; Reuss et al., 1997). The smallest LICs, namely quantum bumps (Wu and Pak, 1975), result from single photon absorptions and the opening of ~15 TRP and TRPL channels, producing peak responses of ~10 pA (Henderson et al., 2000). The influx of Ca^2+^ is particularly important, as it is not only the predominant mediator of the LIC but also a necessary modulator of the whole phototransduction machinery (Hardie, 1991, 1995a; Henderson et al., 2000; Hardie et al., 2001, 2012; Gu et al., 2005; Liu et al., 2008).

It is widely believed that microvillar photoreceptors generally rely on the phosphoinositide cascade (Hardie and Postma, 2009). Therefore, the detailed knowledge about *Drosophila* phototransduction machinery provides a useful basis for gaining insight on how light-induced signals might be generated and regulated in the photoreceptors of other insect species. This question becomes particularly interesting when differences in visual ecology among species are considered. For example, how do the *Drosophila* phototransduction and the signals it produces differ from those in more dark-active species? The American cockroach (*Periplaneta americana*) can be considered as a nocturnal species that shows clear visually guided behavior (Kelly and Mote, 1990; Ye et al., 2003), although light as a sensory modality seems to be of secondary importance for it. The cockroach has relatively large apposition-type compound eyes with unusual structural (Butler, 1971, 1973; Trujillo-Cenóz and Melamed, 1971) and functional (Heimonen et al., 2006) irregularities. The functional variation and signaling properties in cockroach photoreceptors have been suggested to be an optimization for vision in dim light (Heimonen et al., 2006, 2012). Preliminary findings have also implied that the functional variation could originate from phototransduction processes (Heimonen et al., 2012). However, nothing else is really known about the cockroach phototransduction, and a basic description of LICs in cockroach photoreceptors is still lacking.

In this study, we describe the basic properties of cockroach LICs by starting from the level of quantum bumps. We then show the behavior of macroscopic LICs at different light levels, and propose that the response variation found in previous studies may partly stem from variation in rhabdom size among photoreceptors. In addition, we show preliminary results about the effects of manipulation of Ca^2+^ on LIC waveforms, and show that, in contrast to *Drosophila*, the cockroach LIC is only moderately Ca^2+^-selective.

## Materials and methods

### Animals

All experiments were performed on adult male cockroaches (*Periplaneta americana*). Animals were received as adults from Blades Biological Ltd (Cowden, Edenbridge, UK) and were subsequently kept at 25°C under a 12 h day/night rhythm.

### Isolation of ommatidia

Cockroaches were anesthetized for capture with CO_2_. Isolation of ommatidia was done under red light conditions. After removing the body and antennae from the head, one eye was carefully sliced off with a sharp razor blade. The retina was dissected with a flattened insect pin and subsequently cut into several pieces. After an incubation time of ~20 min in extracellular solution complemented with 0.2 mg/ml collagenase type 2 (Worthington Biochemical Corp., Lakewood, NJ USA) and 0.2 mg/ml pancreatin (Sigma-Aldrich), retina pieces were gently triturated until ommatidia separated. Individual ommatidia, obtained after trituration with custom-made glass micropipettes, were allowed to settle in the recording chamber on the stage of an inverted microscope (Axiovert 35 M, Zeiss, Germany).

### Electrophysiology

Patch-clamp recordings were performed as described before (Chyb et al., 1999). Briefly, an Axopatch 1-D amplifier (Molecular Devices, USA) was used; data were digitized and recorded on a laboratory computer using PClamp 10 software (Molecular Devices, USA). Recording electrodes had a resistance of 4–11 MΩ that allowed recordings in the whole cell configuration with an access resistance of <30 MΩ. Access resistance was monitored throughout the experiments. Seal resistance was typically greater than 10 GΩ, membrane resistance in darkness was between 250 MΩ and several GΩ. For most measurements a series resistance compensation of at least 80% was applied. However, during quantum bump recordings, no series resistance compensation was applied, because the recorded currents are then very small. If not stated otherwise, no liquid junction potential (LJP) was corrected (−4 mV). Photoreceptors were clamped to a holding potential of −70 mV. Cells were stimulated with a green (525 nm) light emitting diode (LED) via the fluorescence port of the microscope. Quantum bump experiments were sampled at 2 kHz with either 200 Hz (for steady light) or 500 Hz (for flashes) cut-off of low-pass filtering (80 dB/decade Bessel filter). In 14.5 min bump recordings, 1 kHz sampling and 200 Hz filtering was used. All other experiments were sampled at 2 kHz with 500 Hz or 1 kHz cut-off for filtering.

### Solutions for patch-clamp experiments

Cockroach bath solution contained (in mM): 120 NaCl, 5 KCl, 4 MgCl_2_, 1.5 CaCl_2_, 10 N-Tris-(hydroxymethyl)-methyl-2-amino-ethanesulfoncic acid (TES), 25 proline and 5 alanine, pH was adjusted to 7.15 (NaOH). Nominally Ca^2+^-free bath solution (“0” [Ca^2+^]) contained (in mM): 120 NaCl, 5 KCl, 4 MgCl_2_, 10 TES, 0.5ĖGTA, 25 proline, and 5 alanine, pH 7.15 (NaOH). Intracellular solution contained (in mM): 140 KCl, 10 TES, 2 MgCl_2_, 4 Mg-ATP, 0.4 Na-GTP, and 1 NAD, pH was adjusted to 7.15 (KOH). When 10 mM EGTA was used in the intracellular solution it was loaded with 4 mM CaCl_2_ to obtain ~150 nM and ~6 mM free Ca^2+^ and EGTA, respectively (calculated in Webmaxc Extended by Chris Patton, Stanford University, USA). The intracellular solution for recording of reversal potential (*E_rev_*) under bi-onic conditions contained (in mM): 120 CsCl, 15 TEA-Cl, 10 TES, adjusted to pH 7.15 (N-methyl-D-glucamine, NMDG, which is supposed to be impermeable). The extracellular solution for recording of *E_rev_* of monovalent ions contained (in mM): (1) 130 NaCl, 10 TES, 25 proline and 5 alanine, pH 7.15 (NMDG) for measurement of *E_rev_* for Na^+^.; (2) 130 LiCl, 10 TES, 25 proline and 5 alanine, pH 7.15 (NMDG) for measurement of *E_rev_* for Li^+^; and (3) 130 KCl, 10 TES, 25 proline and 5 alanine, pH 7.15 (NMDG) for measurement of *E_rev_* for K^+^. Extracellular solutions for *E_rev_* of divalent ions were (in mM): (1) 10 CaCl_2_, 120 NMDG-Cl, 10 TES, 25 proline and 5 alanine, pH 7.15 (NMDG) for measurement of *E_rev_* for Ca^2+^; (2) 10 MgCl_2_, 120 NMDG-Cl, 10 TES, 25 proline and 5 alanine, pH 7.15 (NMDG) for measurement of *E_rev_* for Mg^2+^; and (3) 10 BaCl_2_, 120 NMDG-Cl, 10 TES, 25 proline and 5 alanine, pH 7.15 (NMDG) for measurement of *E_rev_* for Ba^2+^. The presence or absence of Cl^−^ in the solutions did not change the properties of the LIC. All chemicals were purchased from Sigma Aldrich unless stated otherwise.

### Analysis of single photon signals

Quantum bump analysis was performed in MatLab (Mathworks, USA), and was mostly based on the analysis by Henderson et al. (2000). Bumps were detected using a preset amplitude threshold. Due to the relatively large size of bumps the threshold was set to a value that would easily stand out from noise (−4 pA). Fused, double peaked bumps were removed. After detecting the location of bump peaks, bump beginnings and ends were determined from the first points around the peaks that were equivalent to two standard deviations of background noise. Bump parameter distributions were fitted with either the normal distribution,

(1) $$P(p)=\frac{1}{\sqrt{2\pi \sigma^{2}}}\exp\left(\frac{-{\left(p-m\right)}^{2}}{2\sigma^{2}}\right),$$

or lognormal distribution,

(2) $$P(p)=\exp\left(\frac{-\ln{\left(p/t_{pk}\right)}^{2}}{2s^{2}}\right),$$

where σ is the standard deviation, *m* is the mean, *p* is the fitted parameter, *t_pk_* is the time-to-peak, and *s* is the skewness.

### Poisson statistics

If a light response consists of discrete responses, the probability *p_n_* for the occurrence of *n* responses per time period will follow the Poisson distribution:

(3) $$p_{n}=\frac{e^{-m}m^{n}}{n!},$$

where *m* is the mean number of responses, i.e., absorbed photons, which can be estimated from the probability of failed absorption (*n* = 0):

(4) $$m=-\ln\left(p_{0}\right).$$

The probability that *n* or more photons are required to elicit a light response follows the cumulative Poisson distribution:

(5) $$p_{\geq n}=\sum_{n=0}^{\infty }\frac{e^{-m}m^{n}}{n!}.$$

By assuming that the dispersion of response size, in this case the response integral, or charge (*Q*), follows the Gaussian distribution, the average size produced by a single photon absorption can be estimated via a sum of Gaussian functions weighted according to the Equation (3):

(6) $$p(Q)=\sum_{n=0}^{\infty }\frac{e^{-m}m^{n}}{n!}\frac{1}{\sqrt{2\pi \left(\sigma_{0}^{2}+n\sigma^{2}\right)}}\exp\left(\frac{-{\left(Q-n\mu \right)}^{2}}{2\left(\sigma_{0}^{2}+n\sigma^{2}\right)}\right)\text{​},$$

where σ_0_ is the standard deviation of failed responses and σ is the standard deviation of single photon response *Q*. μ is the mean *Q* of a single photon response, estimated from relation *Q_a_*/*m*, where *Q_a_* is the overall mean *Q* of failures and successful responses. The Poisson statistics presented in this study are based on (Hecht et al., 1942), (Baylor et al., 1979), and (Henderson et al., 2000).

### Relative permeability

The permeability ratios under bi-ionic conditions were calculated from *E_rev_* of LICs according to (Hille, 2001):

(7) $$P_{M}:P_{Cs}=\frac{{\left[Cs^{+}\right]}_{in}}{{\left[M^{+}\right]}_{out}}\exp\left(\frac{E_{rev}F}{RT}\right)$$

and

(8) $$P_{D}:P_{Cs}=\frac{{\left[Cs^{+}\right]}_{in}}{4{\left[D^{2+}\right]}_{out}}\exp\left(\frac{E_{rev}F}{RT}\right)\left(\exp\left(\frac{E_{rev}F}{RT}\right)+1\right),$$

where *P* stands for permeability, *M* for monovalent ion, *D* for divalent ion, *F* for Faraday constant, *R* for gas constant and *T* for temperature.

### Statistics

Data analysis and statistics were performed with Origin 9 (OriginLab Corp., Northampton, MA, USA) and MatLab (Mathworks, USA). The comparison of Spearman's ρ correlation coefficients, sample normality tests (the D'Agostino normality test) and the Mann-Whitney tests (two-tailed; *p* < 0.05) were performed in Origin 9. Numerical results are presented either as mean ± standard deviation or median followed by interquartile range in brackets (i.e., 1st–3rd quartile).

## Results

The sample of photoreceptors used in this work was not selected in any other way than viability and stability of responses. Therefore, it contains (randomly) many types of dynamics of the light-response (Heimonen et al., 2006).

### Quantum bumps elicited by dim light

**Figure 1:**
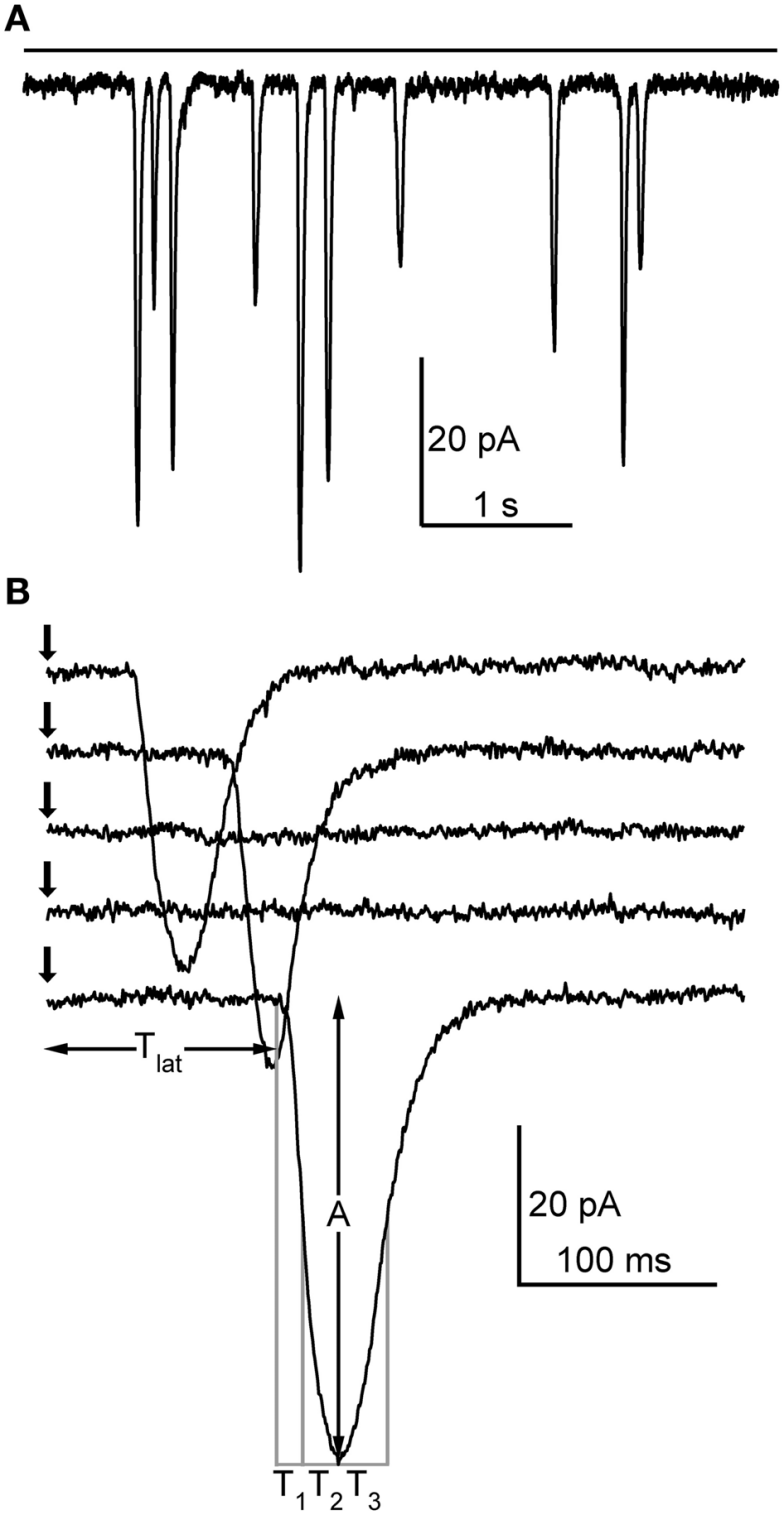
**Elementary light-induced currents (i.e., quantum bumps) in cockroach photoreceptors. (A)** In whole-cell configuration, continuous dim light (horizontal bar; relative intensity 10^−7^) elicited discrete events with variable size. **(B)** Responses to a repeated 1 ms dim flash (arrow). Note how in two traces the flash fails to elicit a response. In this particular cell, the flash was calculated from Equation (4) to contain ~0.27 effective photons on average. The parameters analyzed from the detected quantum bumps are also shown.

Bump-like discrete events with variable amplitudes could be elicited in whole-cell voltage-clamp of cockroach photoreceptors with either continuous light (Figure 1A) or short flashes (Figure 1B), as also described in a recent study of cockroach voltage-dependent currents (Salmela et al., 2012). In order to observe these discrete events with continuous light, the light level was first adjusted with neutral density filters to be low enough not to produce any response. The light level was then increased until discrete inward current jumps could be observed (Figure 1A). Further increase in light intensity increased the frequency of these events until clear summation started to occur, indicating that the responses were indeed bumps. Similarly, when short flashes were used, the light level was increased until it was capable of eliciting a response with ~50% success rate. The flash recordings usually consisted of sets of 1 s recordings repeated 100 times. The health of the cell and recording conditions (resting potential and series resistance) was routinely checked between these sets. Using a similar protocol as in *Drosophila* bump studies (Henderson et al., 2000), the cockroach bumps were analyzed in terms of latency (the time between the flash occurrence and response onset, *T_lat_*), 50% rise time (*T*_1_), 50–100% rise time (*T*_2_), 50% decay time (*T*_3_), and peak amplitude (*A*) (Figure 1B). The *Q* of quantum bumps was also calculated.

### Cockroach quantum bumps conform to poisson statistics

**Figure 2:**
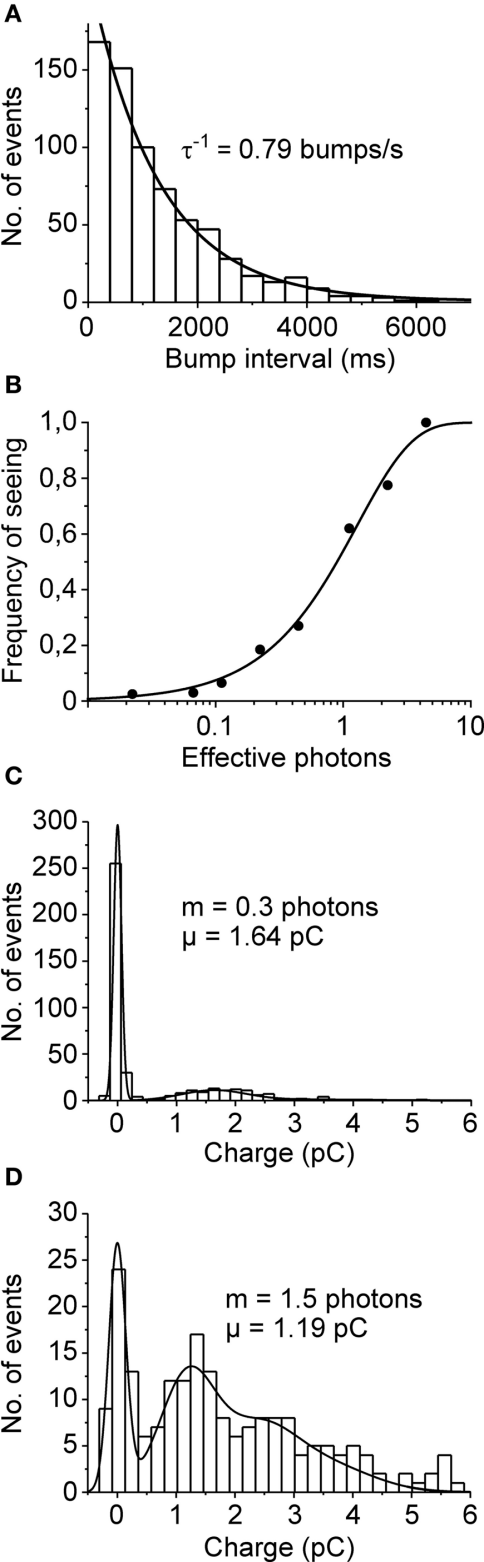
**Poisson statistics of quantum bumps. (A)** Distribution of inter-bump-interval was well-fitted with a single exponential function (time constant was set according to the measured bump rate 0.79 s^−1^; *N* = 689 bumps). **(B)** Frequency of seeing. The proportion of successes, i.e., the probability of detecting a flash is plotted as a function of estimated number of effective photons (data from one cell). The light intensity was changed by adjusting flash duration from 1 to 200 ms. The data is in line with the Poisson prediction assuming that the absorption of one or more photons (*n* = 1) elicits a response. **(C,D)** *Q* distributions of bumps (set to have positive sign) elicited by flashes containing on average **(C)** 0.3 photons (μ = 1.64 pC; *N* = 397) and **(D)** 1.5 photons (μ = 1.19 pC; *N* = 198). The data was collected from two different cells. The smooth curves are fits of Equation (6) to the histogram data.

In order to be considered as single photon absorptions, the cockroach bumps should follow Poisson statistics. First we wanted to see whether the distributions of time intervals between bumps recorded during continuous illumination followed the Poisson prediction (see, e.g., Yeandle and Spiegler, 1973). This could be tested by fitting a single exponential to inter-bump-interval histogram with the reciprocal of the time constant set to the measured bump rate. Figure 2A shows that the cockroach bump intervals indeed follow Poisson distribution. Moreover, if cockroach bumps resulted from the absorptions of at least one photon, the probability of having a successful flash-evoked response should follow Equation (5) with *n* = 1. To test this we made a series of repeated light flash experiments where the intensity of a light flash was varied by changing its duration (the amount of effective photons per flash was estimated by bump calibrations in dim continuous light). Each set of intensities (100–200 repetitions) produced a successful response at a different rate. These rates, or the frequencies of seeing, were then plotted against flash intensities (Figure 2B). On the assumption that one or more photons are required to evoke a response, the data was well-fitted with Equation (5).

The stochastic nature of photon absorption will also result in increased probability of having more than one photon absorptions per flash when intensity is increased. Therefore, based on the Poisson prediction, if *Q* follows Gaussian distribution, the *Q* distribution of all responses should consist of the sum of Gaussian distributions (Baylor et al., 1979). To test this we applied series of short flashes (containing either 0.3 or 1.5 photons on average) and collected the *Q* of both failures and successful responses into a histogram (Figures 2C,D). The results were then fitted with Equation (6), σ being the only free parameter. The sum of Poisson-weighted Gaussian functions fitted the data reasonably well; note that the large dispersion of event size caused a significant group overlap (Figure 2D). Together these results strongly support the notion that the recorded bumps conform to Poisson predictions and represent the elementary responses of cockroach photoreceptors to single photons.

### Analysis of bump parameters

**Figure 3:**
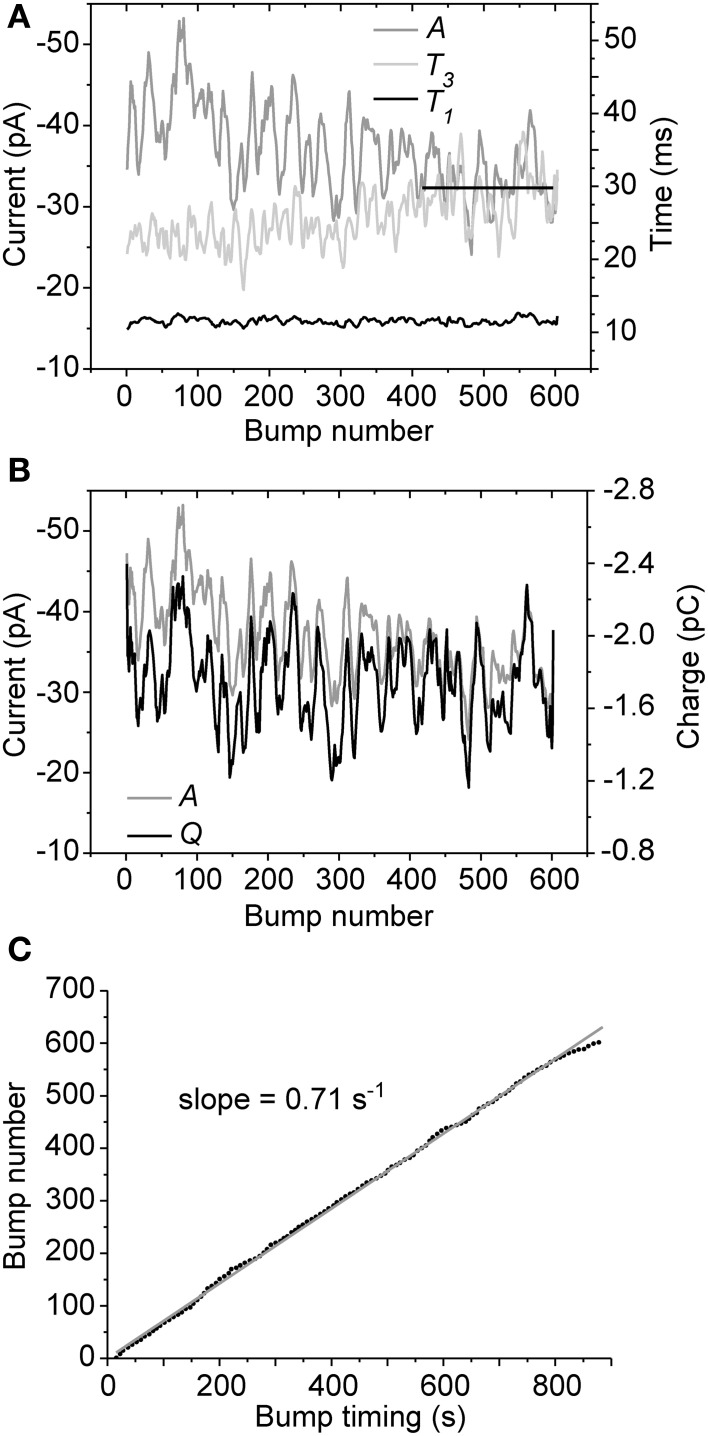
**Bump parameter stability during continuous illumination**. Representative data from a single cell are shown (overall 605 bumps were recorded during a 14.5 min-long light pulse). **(A)** While *T*_1_ remained relatively stable, *A* and *T*_3_ clearly drifted in time until stabilizing within the last minutes of the recording (straight black line). **(B)** Despite the drifting *A*, *Q* remained more or less stable during the recording period. **(C)** Bump occurrence times were well-fitted with a linear function, suggesting that bump rate (light sensitivity) remained relatively unchanged over time. For clarity, the parameters in **(A)** and **(B)** are presented as 20 points moving averages.

We then tested if the occurrence or waveform properties of cockroach bumps change in time by recording responses to 14.5 min-long pulses of dim light. In all cells tested (*N* = 6) both *A* and *T*_3_ had a tendency to drift until reaching a stable level, while *T*_1_ and *Q* remained relatively stable throughout the recording period (Figures 3A,B). Plotting bump peak times together with the running numbers of collected bumps indicated that the bump rate, i.e., light sensitivity, remained roughly the same during the stimulation period (Figure 3C). These results, together with relatively stable resting potential (always below −50 mV) and cell input resistance, suggested that the minor drifting in the observed parameters was not a result of cell rundown. Instead, the results implied that the cells were possibly reaching an equilibrium that might involve mixing of cytosol and pipette solution.

**Figure 4:**
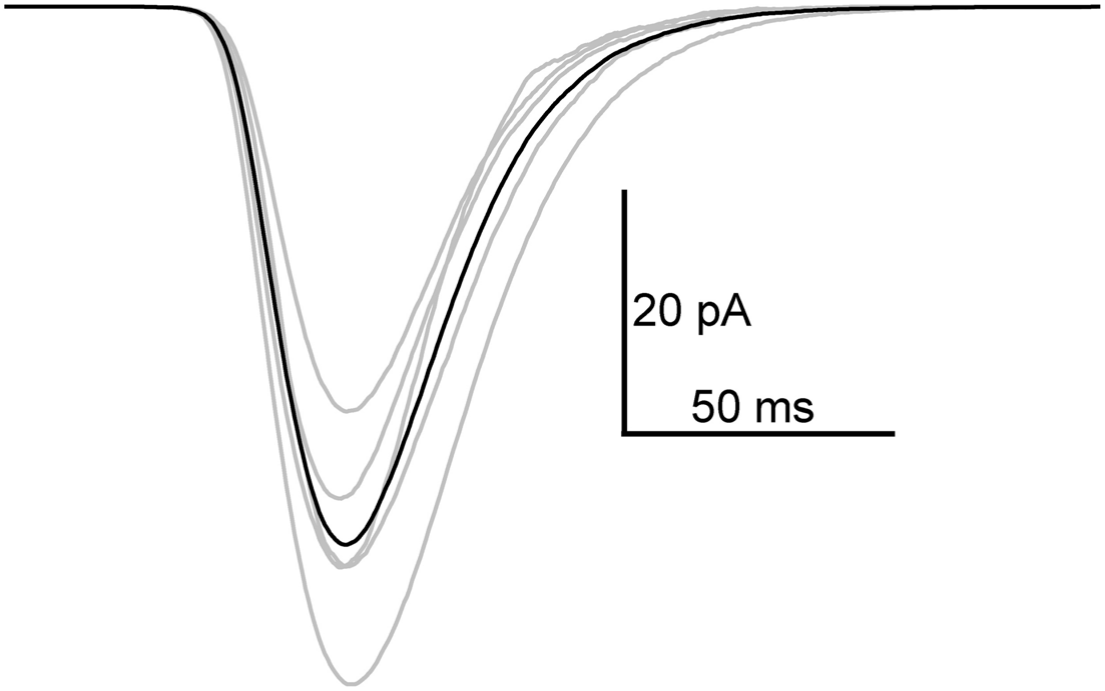
**Average quantum bump in 1.5 mM extracellular Ca^2+^**. The average bump (black) was calculated from average bumps of five cells (gray). Before taking the average in each cell, the original bumps were aligned by parameter *T*_1_. From 73 to 138 bumps were used for averaging.

**Figure 5:**
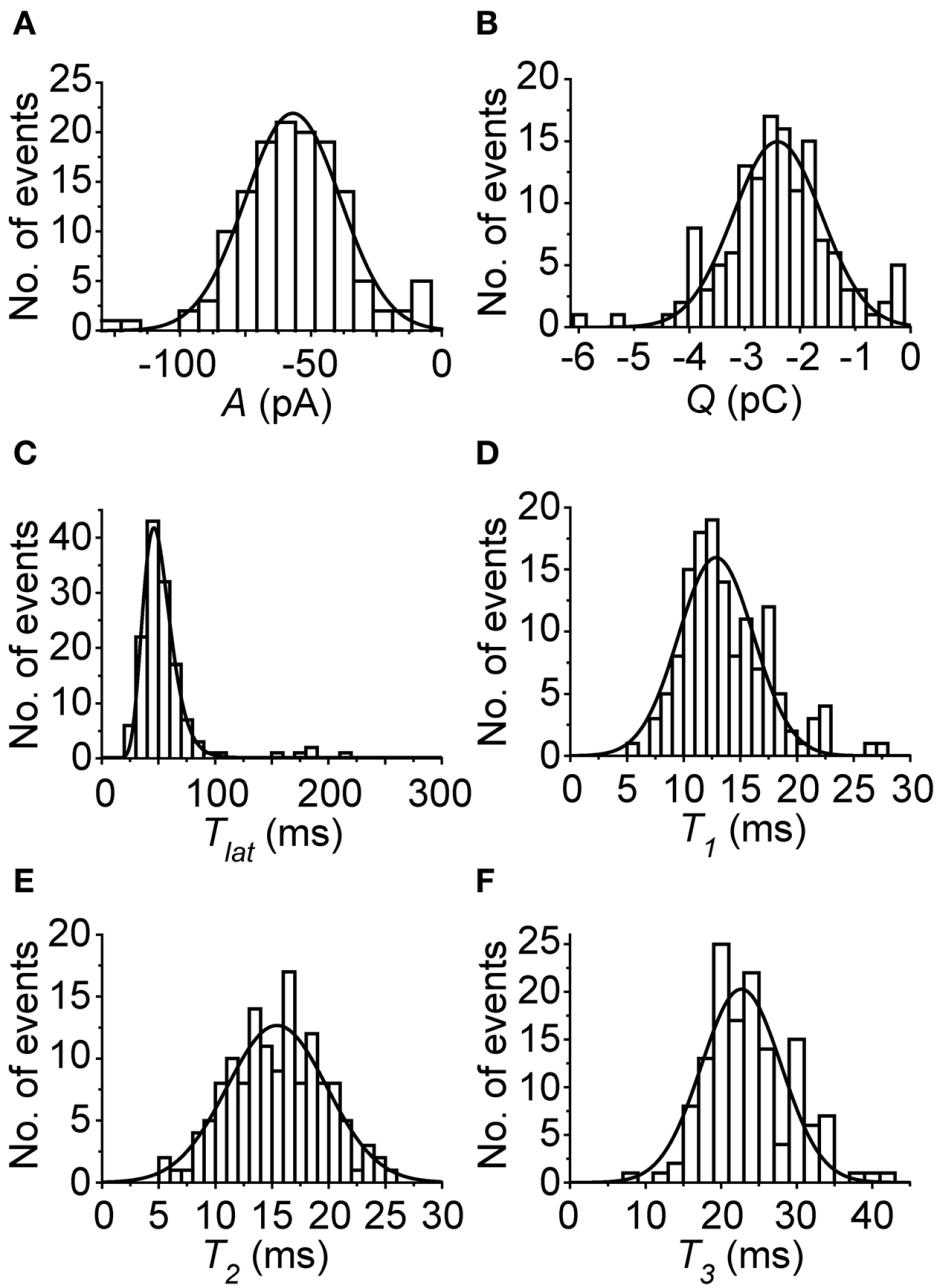
**Representative histograms of bump parameters from one cell in control conditions. (A)** *A*; **(B)** *Q*; **(C)** *T_lat_*; **(D)** *T*_1_; **(E)** *T_2_*; **(F)** *T*_3_. All parameter histograms, except for *T_lat_*, which was fitted with Equation (2), have been fitted with Equation (1). In all cases, the fits were reasonable, and indicate that the parameters were collected mostly from a single group of bumps. However, the bars close to zero in **(A)** and **(B)** suggest that a few false positives, possibly spontaneous G-protein activations, may have been included in the analysis.

Next, flash-induced bumps were more thoroughly analyzed in terms of parameters presented in Figure 1B. However, this time more accurate quality criteria were used to select the bumps. First, the flash intensity was set low enough to maximize the number of single photon absorptions per flash (<50% success rate). Secondly, due to possible drifting of bump waveform, the distribution of *A* had to pass the D'Agostino normality test in order to be accepted for analysis. As a result bumps from five photoreceptors were accepted for analysis. Figure 4 shows the average bump calculated from the averages of the five cells (also shown in light gray traces). The distributions of the analyzed bump parameters indicated that they were mostly from a single class of bumps (Figure 5). The *T_lat_* distribution (Figure 5C) was well-fitted with a lognormal function. Median *T_lat_* across all cells (*N* = 5) was 58 (49–69) ms, and the average *t_pk_* and average half-width (2.35·*t_pk_*·*s*; from Howard et al., 1984), derived from Equation (2), were 53 ± 11 and 16 ± 6 ms, respectively. Table 1 summarizes the results of statistical analysis for other bump parameters. The non-exponential nature of parameter histograms (Figure 5) suggest that the parameters are not produced by a simple 1st order reaction system (Kirkwood and Lisman, 1994). Such a stochastic system would also produce coefficients of variation (CV), i.e., standard deviation divided by mean, close to unity (Henderson et al., 2000). However, CVs in Table 1 indicate that cockroach bump parameters do not vary as much, but differ from those in *Drosophila* (Henderson et al., 2000).

**Table 1:** **Statistics of bump parameters under control conditions (1.5 mM Ca^2+^; *N* = 5 cells)**.

	***A* (pA)**	***T*_1_ (ms)**	***T*_2_ (ms)**	***T*_3_ (ms)**	***Q* (pC)**
Mean	−46.3	13	13	20	−1.69
STD	19.3	5	4	7	0.83
CV	0.42	0.38	0.31	0.35	0.49

*The number of analyzed bumps in each cell varied from 73 to 138. Abbreviations: STD, standard deviation; CV, coefficient of variation (STD/Mean)*.

Interdependence of bump generation processes can be assessed by examining correlation coefficients between bump parameters (see, e.g., Howard, 1983; Henderson et al., 2000). Table 2 shows the values of Spearman's ρ calculated for two representative cells. Although many different small but statistically significant correlations were found (e.g., between *T_lat_* and *T*_1_) in the five analyzed cells, the only correlations showing consistency were those between *A* and *T*_3_ (ρ = 0.17–0.47), and *T*_2_ and *T*_3_ (ρ = 0.26–0.46). These results suggest that at least *T_lat_* and bump waveform (including *A*) are generated by processes independent of each other.

**Table 2:** **Correlation (Spearman's ρ) between bump parameters under control conditions**.

	***A***	***T_lat_***	***T*_1_**	***T*_2_**	***T*_3_**
**CELL 1**					
*T_lat_*	0	–			
*T*_1_	0	0	–		
*T*_2_	0	−0.21^*^	0	–	
*T*_3_	0.17^*^	0	0	0.45^*^	–
**CELL 2**					
*T_lat_*	0	–			
*T*_1_	0.27^*^	−0.29^*^	–		
*T*_2_	0.25^*^	0	0	–	
*T*_3_	0.37^*^	0	0	0.46^*^	–

*Two out of five cells are presented. A were set to have positive sign. The number of bumps was 138 and 112 for Cell 1 and Cell 2, respectively*.

* *p < 0.05, two-tailed*.

### Macroscopic light-induced currents

**Figure 6:**
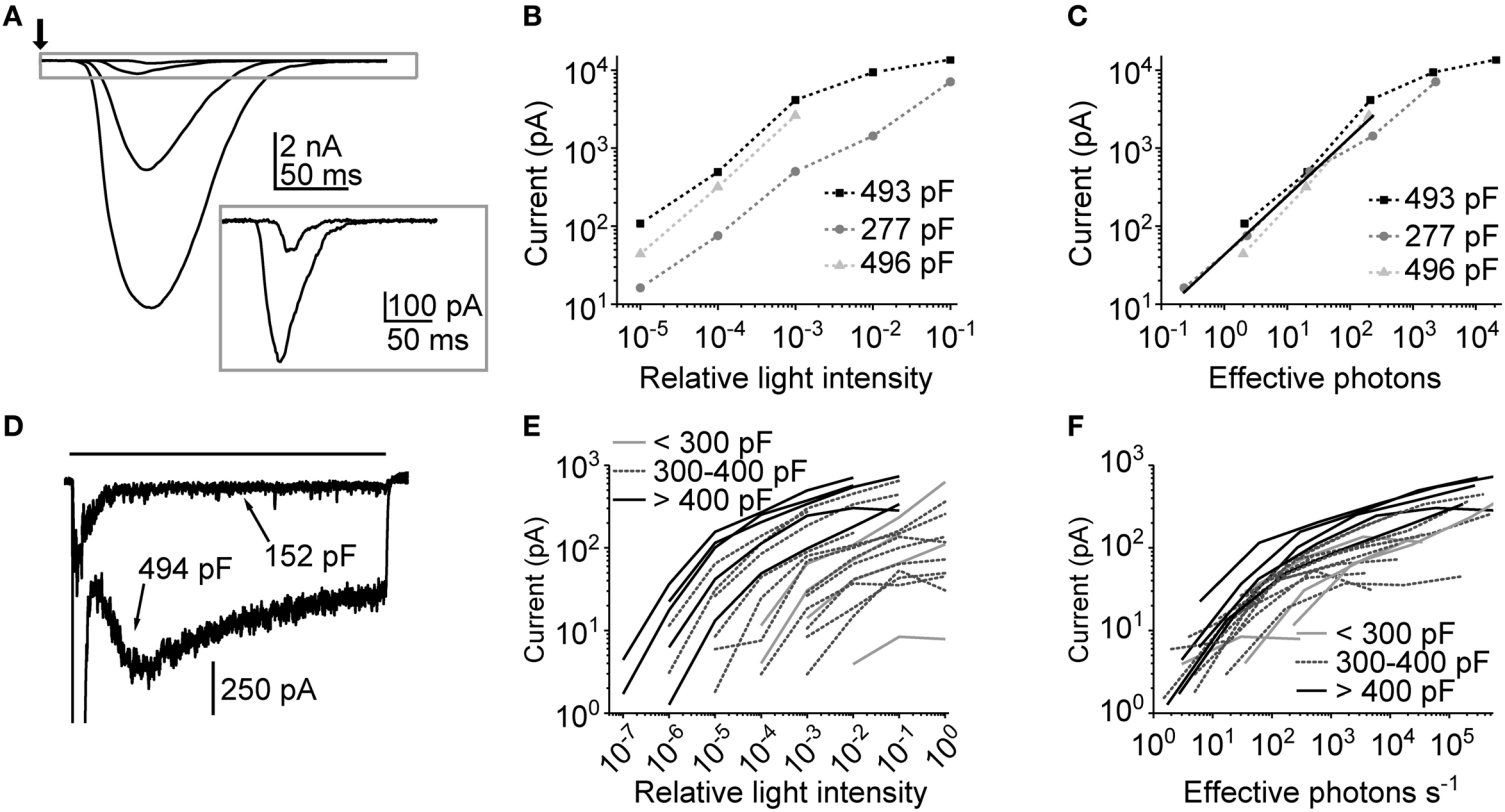
**Macroscopic light-induced currents in cockroach photoreceptors. (A)** An example of responses to a 10 ms flash (arrow) with increasing intensity (from 210 to 210,000 effective photons/s, corresponding to relative intensities of 10^−5^–10^−2^). The inset shows the two smallest LICs in a different scale. **(B)** Flash response amplitudes of three cells plotted as a function of relative flash intensity. **(C)** Flash response amplitudes of the same cells as a function of effective photons per second (i.e., number of bumps per second). The linear fit represents linear growth of response amplitude with a slope of 5.7 pA/effective photon. **(D)** Examples of responses to a 10 s light pulse (black bar) from two cells with indicated membrane capacitances. For clarity only one light level is shown (relative intensity 10^−2^). **(E)** Average plateau currents (calculated from the 7 s stretch 2 s after the onset of 10 s light pulse) plotted as a function of relative light intensity (*N* = 21 cells). The cells have been sorted into three arbitrary groups by their capacitance. **(F)** Average plateau currents from the same cells as a function of effective photons per second. The photon calibration was carried out by calculating the number of bumps during a 30 s dim light pulse.

Thus, far, macroscopic LICs in cockroach photoreceptors have been briefly described in two studies from our group (Heimonen et al., 2012; Salmela et al., 2012). In the latter study, the major finding regarding LICs was that the functional variation could stem from differences in phototransduction processes among photoreceptors (Heimonen et al., 2012). However, it remained unclear what are the processes involved, and how the LICs would behave at different light levels. To answer those questions we recorded LICs at several light levels by using both flashes (10 ms) and pulses (10 s) of light. Our previous study had indicated that the electrical properties, such as membrane capacitance, tend to vary a lot among cockroach photoreceptors (Salmela et al., 2012). Therefore, membrane capacitances were also determined for each cell. Figure 6A shows representative LICs recorded from a 493 pF cell. Figure 6B shows the amplitudes of the LIC in the same cell, together with results in two other cells, as a function of relative light intensity. The behavior of LIC amplitudes suggests that the large capacitance cells had higher light sensitivity than their small capacitance counterpart. However, once the light intensities were adjusted to match the number of effective photons (estimated from bump calibrations), the LIC amplitudes fell into a same range (Figure 6C). A rough estimation suggests that the LIC amplitudes increase linearly up to a range of 1–3 nA and 200–300 effective photons, with linear relation of 5.7 pA/effective photon.

As it was expected from earlier studies (Heimonen et al., 2006, 2012), the use of 10 s light pulses resulted in highly variable waveforms of LICs across cells. Figure 6D shows examples of two extreme shapes of LICs evoked by the identical 10 s light pulse. This seems to suggest that there could be a link between the LIC response size and the photoreceptor capacitance. In Figure 6E the average plateau responses (time interval between the 3rd and 8th s of a response) of LICs recorded in 21 cells are plotted against relative light intensity. First, it can be seen that the amplitudes span up to seven orders of magnitude of relative light intensities, indicative of high variability in light sensitivity across cells. Secondly, the grouping of cells by their capacitance suggests that capacitance positively correlates with light sensitivity. Again, after adjusting the light intensities according to bump calibrations (this time in terms of effective photons s^−1^), the amplitudes narrowed down into a tighter group, which indicates that, except for the difference in sensitivity, the cells are functionally similar (Figure 6F). This conclusion was supported by finding that the size of quantum bumps did not seem to correlate with cell capacitance (Spearman's ρ = 0; *p* = 0.62; *N* = 5). In conclusion, since capacitance is an indirect measure of membrane area, the sensitivity differences may arise from differences in the size of rhabdomeres.

### Dependence on Ca^2+^

**Figure 7:**
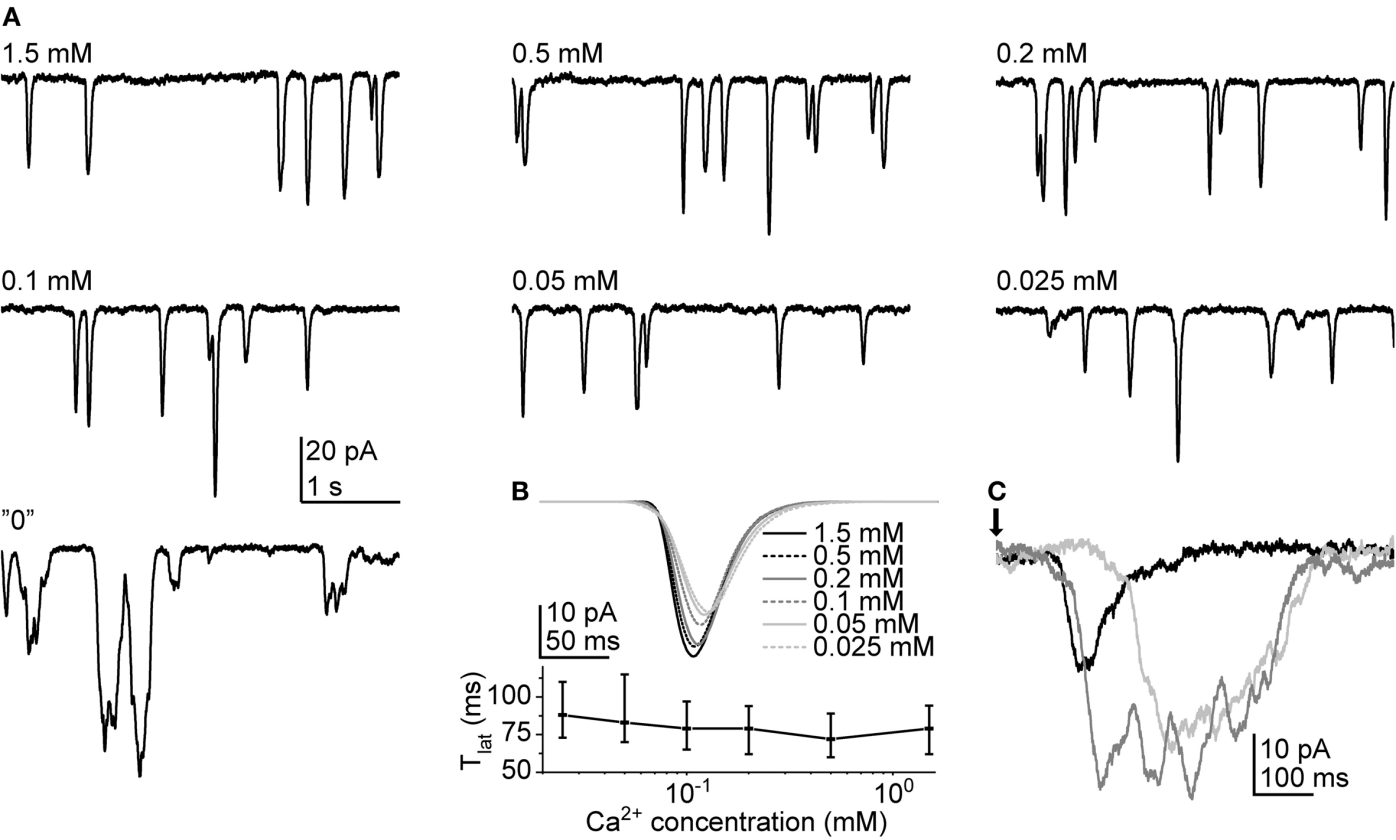
**Quantum bumps at different extracellular Ca^2+^ concentrations. (A)** Quantum bumps recorded during continuous dim light stimulation in the same cell. Conspicuous differences were observed only in “0” [Ca^2+^]. **(B)** Above panel: average flash-induced bumps in the same cell at different Ca^2+^ concentrations. Lower panel: median *T_lat_* as a function of Ca^2+^ concentration. The error bars depict interquartile range. **(C)** Samples of flash-induced bumps in “0” Ca^2+^. The arrow indicates the time of a 1 ms flash.

To see how cockroach LIC is dependent on Ca^2+^ we first tested the effect of a series of different extracellular Ca^2+^ concentrations on cockroach bumps. The test was performed with both continuous light (30 s light pulse) and 1 ms flashes in one cell. The concentration series used consisted of 7 different solutions covering a range from 1.5 mM to nominally Ca^2+^-free (“0” [Ca^2+^]; no added Ca^2+^ plus 500 μM EGTA). The concentrations were chosen following the study by Henderson et al. (2000). Surprisingly, the lowering of extracellular [Ca^2+^] had relatively weak effects on the bump waveform (Figure 7). The bumps induced by a 30 s pulse started to show dramatic effects only at “0” [Ca^2+^] (Figure 7A). With “0” [Ca^2+^] the bump waveforms became substantially slower and prolonged, causing extensive response fusion. Bumps elicited by repeated flashes behaved in a similar manner. The comparison of average bumps (calculated from 65 to 192 bumps) indicated that in Ca^2+^ concentrations from 1.5 mM down to 25 μM the bumps became only slightly smaller and slower (Figure 7B). Although *T_lat_* increased in lower Ca^2+^ concentrations, the changes were relatively small. In “0” [Ca^2+^], the bump shapes were highly unpredictable, varying from smaller rapid events to multi-peaked rectangular-like responses (Figure 7C). Therefore, the analysis of bump waveform parameters was not considered viable in “0” [Ca^2+^]. However, the comparison of averages across three cells suggested that bump *A* did not drop substantially even in “0” [Ca^2+^] (−35.3 ± 17.7 pA in control vs. −30.0 ± 15.7 pA in “0” [Ca^2+^]; *N* = 65–185 bumps). The comparison of samples within each cell indicated a statistically significant difference only in one out of three cases. Another observation made in the same cells was that the *T_lat_* dispersion appeared to increase when Ca^2+^ was omitted. The difference in median *T_lat_* (cell 1: 43 (37–49) ms; cell 2: 45 (36–55) ms; and cell 3: 79 (63–96) ms under control conditions vs. cell 1: 53 (44–68) ms; cell 2: 59 (47–71) ms; and cell 3: 104 (89–162) ms in “0” [Ca^2+^]) was statistically significant within all three cells (*p* < 0.001; *N* = 65–185 bumps). In addition, the half-widths of lognormal fits of the *T_lat_* distributions indicated that Ca^2+^ removal increased the dispersion of *T_lat_* (13 ± 5 ms in control vs. 23 ± 12 in “0” [Ca^2+^]; *N* = 3).

**Figure 8:**
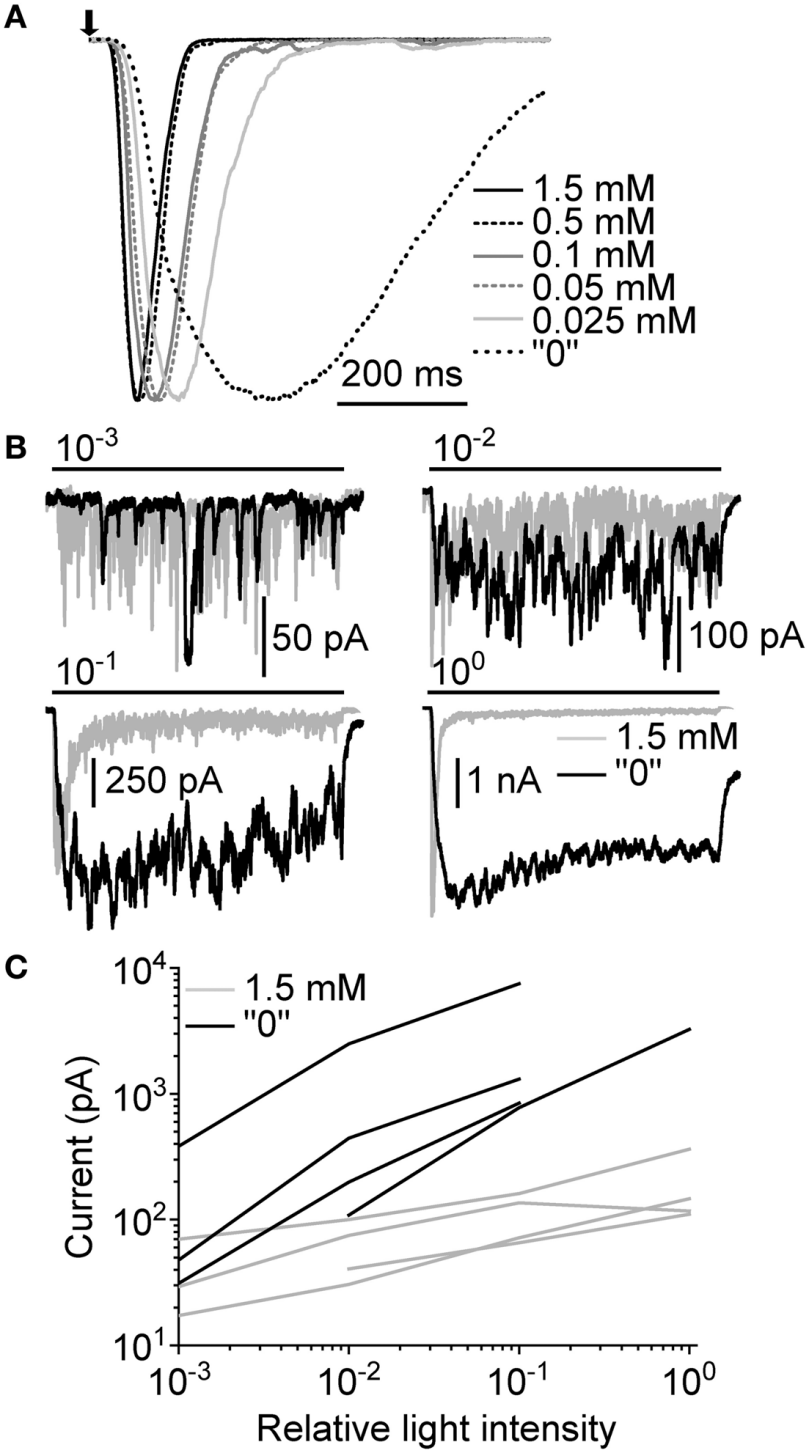
**Ca^2+^-dependence of macroscopic light-induced currents. (A)** Normalized responses to a 10 ms flash (arrow) at different Ca^2+^ concentrations. The flash was estimated to contain ~200 effective photons over the whole concentration range. **(B)** Responses to a 10 s light pulse (bars) at 1.5 mM and “0” extracellular Ca^2+^. Relative light intensities are indicated above the light stimulus bars. **(C)** Comparison of average plateau currents (10 s light pulse) of four cells at 1.5 mM and “0” [Ca^2+^] plotted as function of relative light intensity.

The effects of extracellular Ca^2+^ manipulations were then tested with macroscopic LICs. First, the same set of Ca^2+^ concentrations as above was used with responses to a 10 ms flash in one cell (Figure 8A). As with bumps, the lowering of extracellular Ca^2+^ concentration resulted in only moderately slowed responses (a result of slower bumps and/or the increased dispersion of bump *T_lat_*) until “0” [Ca^2+^] was used. In “0” [Ca^2+^], the LIC amplitude (from ~4 to ~10 nA) and duration increased dramatically. The second test involved using 10 s light pulses at different light levels before and after omission of extracellular Ca^2+^. The use of longer light pulses allowed us to observe changes beyond transient responses, and see how extracellular Ca^2+^ affects light adaptation. Since bump discrimination during continuous stimulation was practically impossible we had to rely on comparing effects using relative light intensities. The omission of extracellular Ca^2+^ resulted in an increase of amplitude and slightly prolonged duration of the LIC when compared to 1.5 mM Ca^2+^ (Figure 8B). The peak-to-plateau transition in the late phase of the LIC was also completely suppressed. However, response delay and the early onset of initial transient were almost as fast in “0” [Ca^2+^] as in 1.5 mM Ca^2+^. The effects were similar in other cells recorded, and the average plateau currents were systematically increased in “0” [Ca^2+^] (Figure 8C). This result suggests that Ca^2+^ influx may be necessary for proper response deactivation.

**Figure 9:**
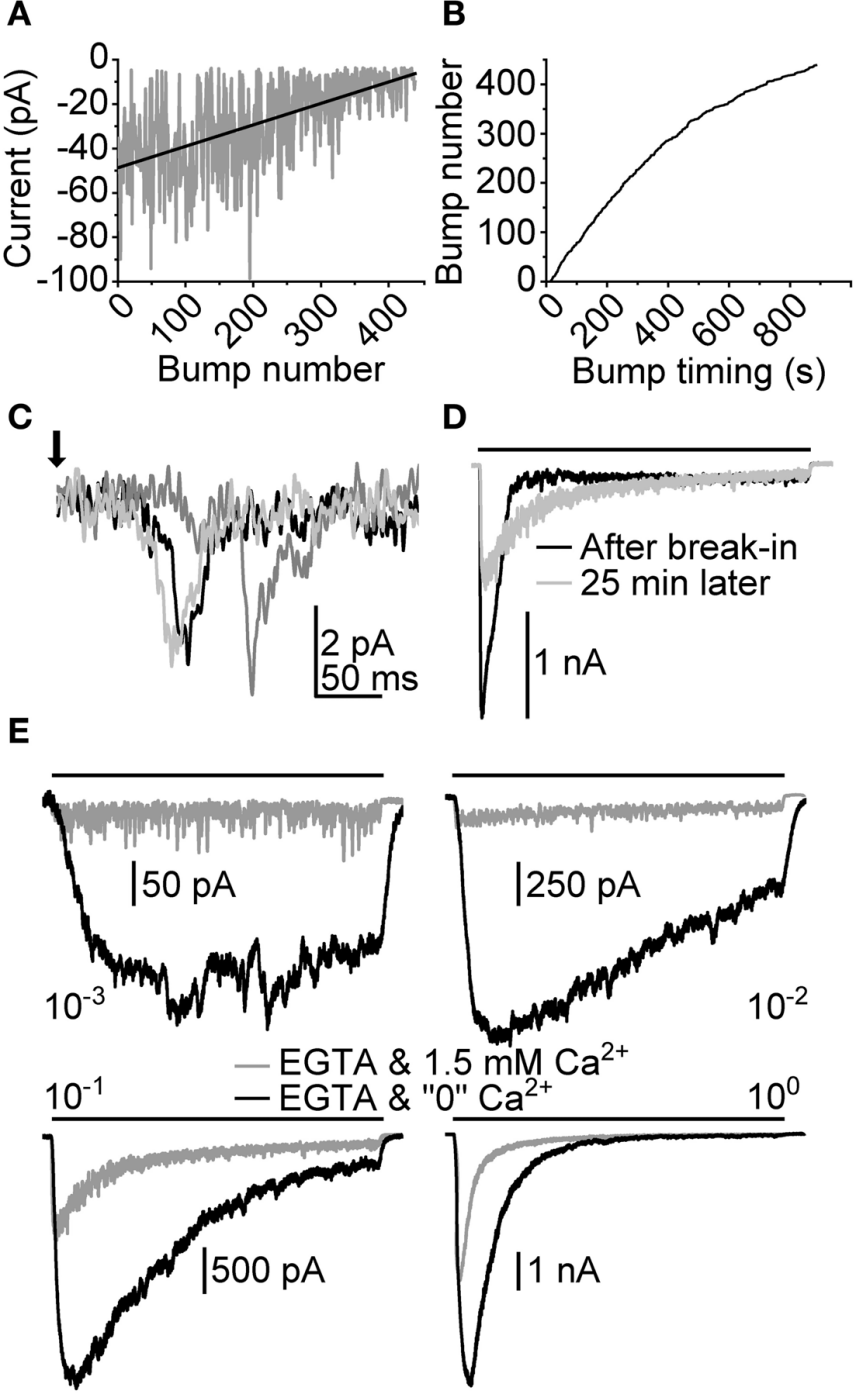
**Effects of intracellular Ca^2+^ chelation on light-induced currents**. Intracellular Ca^2+^ was chelated using 10 mM EGTA (~6 mM free EGTA) in pipette solution. **(A)** Peak amplitudes of bumps (*A*) collected from a recording made with 870 s (14.5 min) continuous dim light after achieving whole-cell configuration. The linear fit depicts the slow deterioration of bump *A* in time. **(B)** Bump occurrence times obtained from the same recording as in **(A)**. The declining tail of the plotted trace indicates a gradual decrease of bump appearance frequency (i.e., light sensitivity). **(C)** Illustration of effects of EGTA on flash-induced bumps. The bumps were recorded ~30 min after establishing whole-cell configuration. **(D)** Effects of 10 mM EGTA on a macroscopic response to a 10 s light pulse (bar; relative intensity 10^−1^). **(E)** Responses to a 10 s light pulse at 1.5 mM and “0” [Ca^2+^] while the pipette solution is loaded with 10 mM EGTA.

To learn more about the possible mechanisms involved in the activation of LICs we also attempted to manipulate intracellular Ca^2+^ by buffering with EGTA. Since EGTA is a relatively slow, millisecond scale Ca^2+^ chelator, its presence in the pipette solution should still allow transient increases in Ca^2+^, e.g., through light-gated channels in microvilli (Henderson et al., 2000). Therefore, in principle, if capacitive Ca^2+^ entry (i.e., the activation of InsP_3_ or ryanodine receptors) was necessary for response excitation in the cockroach, EGTA should be able to inhibit the activation of the LIC. The effects of 10 mM EGTA were first tested on bumps. Figure 9A shows the behavior of bump *A* over a 870 s (14.5 min) light stimulation period started right after achieving the whole-cell configuration. Visual examination suggests that after 100 bumps *A* started to deteriorate. The frequency of the appearance of bumps also decreased with time, which can be seen from the sub-linear relation of bump number with time (Figure 9B).

Flash-induced bumps recorded subsequently in the same cell diminished to barely detectable levels (Figure 9C). This resulted in a relatively poor signal-to-noise ratio, which is why quantitative bump parameter analysis was not carried out in full. *T_lat_* from 72 events were collected, resulting in a median of 84 (66–99) ms. However, although showing otherwise similar behavior with EGTA, bumps recorded in another cell resulted in much shorter *T_lat_*, with median of 38 (34–45) ms (*N* = 110), making definitive conclusions about the effects of EGTA on transduction impossible. We then tested how a prolonged exposure to intracellular EGTA would affect the waveform of the macroscopic response. This was done by first recording a LIC induced by a relatively bright 10 s light pulse right after formation of the whole-cell recording configuration. After 25 min:s the same light pulse was repeated. The resulting LIC showed a clear drop in transient amplitude, and was reminiscent of a response recorded with lower light intensity in control conditions. This behavior was in line with the drop in quantum efficiency observed with bumps recorded in the presence of EGTA. In the same cell, the effects of extracellular Ca^2+^ removal were also tested (Figure 9E). Two notable observations were made. First, the responses in “0” [Ca^2+^] were very much like those detected without EGTA in the pipette (compare to Figure 8B). Secondly, the responses showed conspicuous inactivation in relatively bright light, suggesting the exhaustion of a transduction component.

### The Ca^2+^-selectivity of cockroach light-induced current

**Figure 10:**
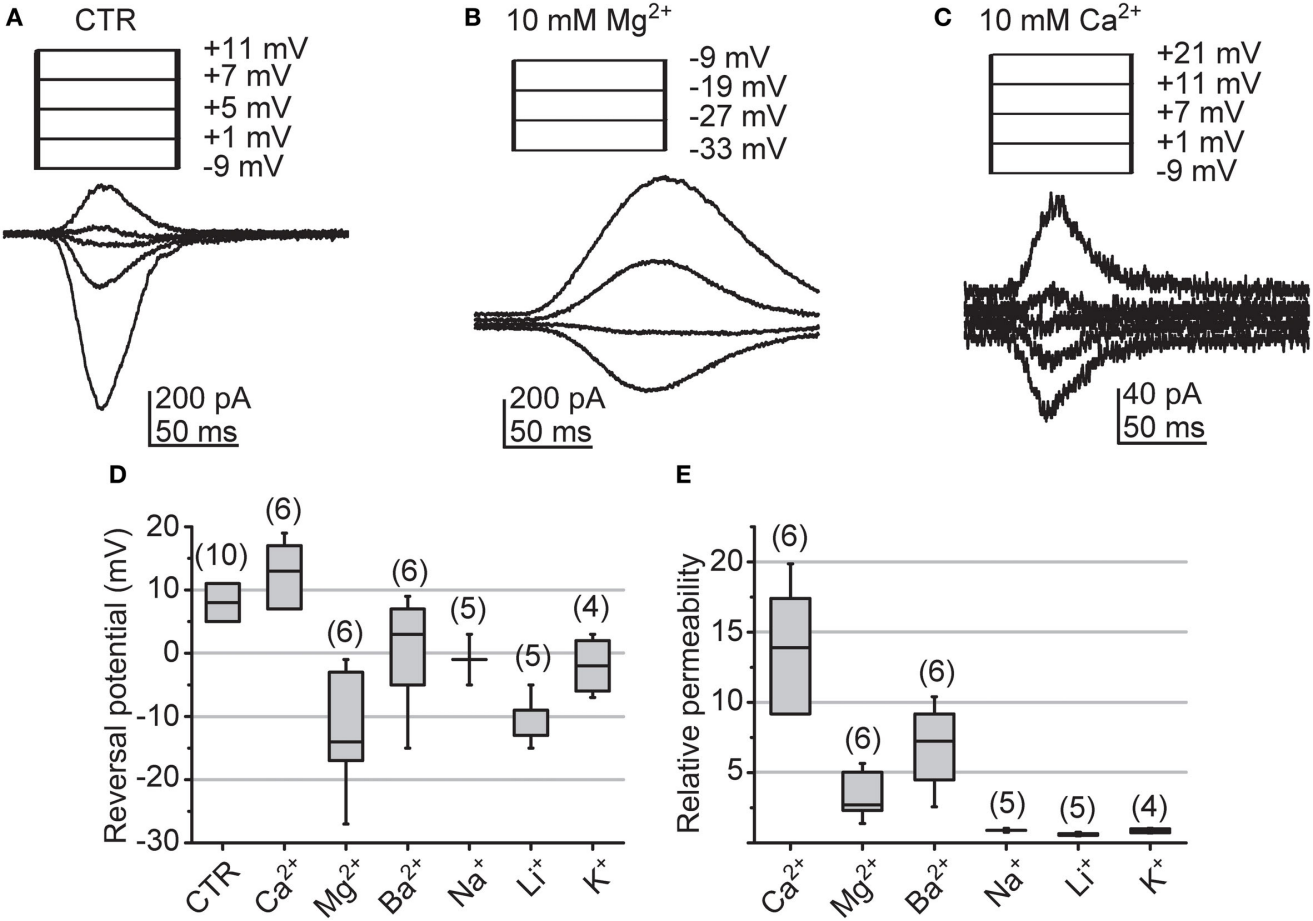
**Reversals of light-induced currents and the relative ionic permeabilities of the associated channels. (A–C)** *E_rev_* of LICs in a single representative cell determined under bi-ionic conditions with 120 mM intracellular Cs^+^ and either normal cockroach bath solution (CTR), 10 mM Mg^2+^, or 10 mM Ca^2+^ in bath (as indicated). Responses were elicited with 50 ms sub-saturating light flashes and stepping the holding potential within a desired voltage interval (from −24 to 16 mV in Ringer; from −20 to 20 mV in Ca^2+^; from −40 to 0 mV in Mg^2+^; all with 2 mV increments). For clarity representative traces around the *E_rev_* and the corresponding voltages are shown. Values are corrected for the LJP. **(D)** Statistics of measured *E_rev_* with CTR and under bi-ionic conditions. The box-plot shows the median (horizontal line in the box), the 1st and 3rd quartiles (the ends of the boxes) and the minimum and maximum values (whiskers). **(E)** Statistics of the relative permeabilities for Ca^2+^, Mg^2+^, Ba^2+^, Na^+^, Li^+^ and K^+^; box-plot as in **(D)**. The numbers presented above each box represent the number of analyzed cells. The permeabilities were calculated according to Equations (7) and (8). Concentration of all other ions as explained in the Methods.

The surprisingly large size of cockroach quantum bumps in “0” [Ca^2+^] suggests that the LIC may not be dependent in the influx of Ca^2+^ to such an extent as in the fruitfly photoreceptors. To estimate the Ca^2+^-selectivity of cockroach light-gated channels we determined the relative ionic permeabilities from LIC *E_rev_* recorded under bi-ionic conditions. The permeabilities of extracellular cations were assessed relative to an intracellular cesium solution, which also contained 15 mM TEA-Cl to attenuate voltage-dependent potassium conductances. The approach was largely adopted from the study by Reuss et al. (1997). The *E_rev_* were determined from LICs induced by a 50 ms flash, while the cell was clamped to different voltages in 2 mV increments (Figures 10A–C). The median *E_rev_* measured under control conditions from 10 cells was 8 (5–11) mV (Figure 10D). The calculation of relative mono- and divalent cation permeabilities according to Equations (7) and (8) revealed only slight selectivity over other cation species for Ca^2+^ (Figure 10E). The median permeabilities relative to Cs^+^ for Ca^2+^ and Na^+^ were 13.9 (9.2–17.4) and 0.9 (0.9–0.9), respectively, giving a ~16:1 selectivity for Ca^2+^ over Na^+^.

## Discussion

Until now our knowledge of phototransduction mechanisms in invertebrate photoreceptors have relied on a few prototypical species. In particular, insect phototransduction has been studied extensively in the fruitfly (*Drosophila melanogaster*) photoreceptors (Hardie and Minke, 1992; Hardie and Raghu, 2001; Hardie and Postma, 2009). However, significant species-specific differences could exist, suggested by work done, e.g., in the honeybee (Baumann and Walz, 1989; Ziegler and Walz, 1990; Walz et al., 1994, 1995), the cockroach (Heimonen et al., 2006, 2012; Salmela et al., 2012), the stick-insect (Frolov et al., 2012), and the field cricket (Frolov et al., 2014). Those differences may be suggested to be linked to different visual ecology of those animals vs. the fruitfly. In this work, we have characterized the LICs in the photoreceptors of the American cockroach, with emphasis on the quantitative analysis of quantum bumps. The study was carried out by using whole-cell voltage-clamp, which allowed the uncoupling of LICs from other major ionic currents, such as voltage-gated K^+^ currents. We also addressed the question how cockroach LIC is dependent on Ca^2+^ by manipulating both extra- and intracellular Ca^2+^ during experiments. Our findings gave us clues about the possible mechanisms responsible for the generation of LIC. Most importantly, we were able to find out that the light-gated channels of cockroach photoreceptors are only moderately Ca^2+^-selective. This finding can be compared to the about two-times more Ca^2+^-selective LIC in *Drosophila* photoreceptors (Reuss et al., 1997). Although the solutions we used were not identical to what was used in that work, that did not appear to compromise photoreceptor viability or their ability to respond to light.

The comparison of bump statistics reveals one conspicuous difference between the cockroach and *Drosophila*: the cockroach bump *A* is ~5 times higher (see Table 1 and Henderson et al., 2000). Large bumps have been recorded also in other nocturnal species (Frolov et al., 2012, 2014), supporting the theory that large bump size is an adaptation for vision in dim light (Warrant and Dacke, 2011) in order to increase signal-to-noise ratio at single photon level. However, although the *T_lat_* and kinetics of cockroach bumps are slightly slower than in *Drosophila*, they still have very similar time-scales. It is therefore highly plausible that the same basic transduction machinery (i.e., PLC-PIP_2_ cascade) is responsible for the generation of cockroach light responses. Accordingly, large bump *A* could be a result of—compared to *D. melanogaster*—a larger number of light-gated channels, a higher single channel conductance, or both.

The lack of systematic statistical dependence between *T_lat_* and bump waveform in cockroach (Table 2) suggests that they are defined by independent mechanisms (Howard, 1983; Keiper et al., 1984; Henderson et al., 2000). In principle, this finding is in conflict with the slowing of *T_lat_* and bump kinetics in “0” [Ca^2+^]. However, the longer *T_lat_* could also stem from changes in the resting levels of cytosolic Ca^2+^ inflicted by the Na^+^/Ca^2+^-exchanger (Hardie, 1996; Gu et al., 2005). Such changes may influence the activity of crucial transduction components, such as PLC (Running Deer et al., 1995). Interestingly, except for one cell, the bump half-width (*T*_2_ + *T*_3_) also showed some correlation with bump *A* (from 0.3 to 0.4). According to the bump scaling theory of Burton (Burton, 2006), which states that bump *A* decreases with decreasing duration, this correlation would be expectable. However, the relatively weak correlation also highlights the considerable variation in bump generation processes. Nevertheless, it would be interesting to find out whether larger bumps could be generated simply by slowing down the relevant enzymatic phototransduction reactions.

From flash-induced macroscopic LICs (Figures 6A–C) we observed a near linear increase in peak amplitude as the light levels were increased up to a point of <1000 effective photons. At this point the currents were already in the range of a few nA where significant series resistance error can be expected. Nevertheless, within the seemingly linear intensity range cockroach LIC increased with a slope of 5.7 pA per effective photon, meaning that the gain of phototransduction is about two times higher than that in *Drosophila* (Henderson et al., 2000). The linear range also corresponds well with the findings relating to information processing in cockroach photoreceptors, which does not seem to increase beyond the intensity of ~1000 photons/s (Heimonen et al., 2012). Another interesting observation was the tendency of high capacitance cells to be more sensitive to light (Figures 6B,E). Since bump size did not correlate with capacitance, this behavior may originate from differences in the number of microvilli in each photoreceptor. Relatively speaking, a lower number of microvilli would not only more easily make a photoreceptor less light sensitive but also render it refractory as the light levels are increased (Figure 6D; see also Song et al., 2012). This type of behavior might also explain the presence of “hyperadapting” cells in the cockroach (Heimonen et al., 2006, 2012), reminiscent of the *Drosophila trp* phenotype (Minke, 1982). Therefore, instead of differences in phototransduction (Heimonen et al., 2012), the functional differences among cockroach photoreceptors may be explained by differences in the size of rhabdomeres and the number of microvilli. This complements the previously reported high functional and structural variability of cockroach photoreceptors (Heimonen et al., 2006).

In *Drosophila* photoreceptors, the importance of Ca^2+^ influx during a light response is undisputed, as it both regulates response amplification and inactivation (Hardie, 1995a), and is also the major mediator of light adaptation (Hardie et al., 2001; Gu et al., 2005; Liu et al., 2008). Clear effects are manifested already at the level of single photon bumps, when the extracellular concentration of Ca^2+^ is lowered to 200 μM (Henderson et al., 2000). Surprisingly, cockroach bumps were not as sensitive to extracellular Ca^2+^ manipulations, and retained their waveform relatively well down to a concentration of 25 μM (Figures 7A,B). Only after the omission of extracellular Ca^2+^ by using a chelator, the cockroach bumps were considerably changed, their waveforms becoming highly unpredictable (Figure 7C). Even then the amplitudes did not deteriorate as dramatically as in *Drosophila* (Henderson et al., 2000). As it would be expected from the convolution of wide-shaped bump and *T_lat_* distribution, the flash-induced LICs behaved similarly as bumps with the same set of Ca^2+^ concentrations (Figure 8A)—only in “0” [Ca^2+^] was the response waveform changed dramatically. With 10 s light pulses the most notable finding was the failure of cockroach LICs to develop the distinctive peak-plateau transition phase in “0” [Ca^2+^] while higher light intensities were used (Figures 8B,C). Basically, this meant that without Ca^2+^ the photoreceptors lost some of the Ca^2+^-dependent negative feedback mechanism(s). One possible candidate could be the Ca^2+^-dependent inactivation of metarhodopsin (Liu et al., 2008). In *Drosophila trp* mutants lacking the Ca^2+^-selective TRP channels, the loss of Ca^2+^ influx may result in PIP_2_ depletion due to uncontrolled hydrolysis by PLC (Hardie et al., 2001). With longer lasting light stimuli this results in transient responses that eventually drop down to baseline level while the light is still on. Accordingly, the removal of extracellular Ca^2+^ should produce a similar effect. Apparently, the use of Ca^2+^-free solutions was not enough in the case of cockroach, as all four cells tested responded robustly at all light levels without showing any indication of LIC rundown (Figures 8B,C). In fact, in three out of four cells, the brightest light level could not be registered anymore as the currents could easily saturate the amplifier.

Assuming that the influx of Ca^2+^ via light-gated channels takes place in a microsecond timescale, free intracellular EGTA should not be able to interfere with the generation of bumps and transient LICs considerably (Hardie, 1995b; Henderson et al., 2000). Interestingly, EGTA did not only diminish cockroach bump *A*, but simultaneously reduced sensitivity to light as well (Figures 9A–C). However, intracellular EGTA did not prevent photoreceptors from producing prominent macroscopic LICs (Figures 9D,E), suggesting that chelation reduces the response size only by impeding intracellular Ca^2+^ accumulation and thus proper Ca^2+^ feedback. It should be also noted that in these experiments, the pipette solution was loaded with Ca^2+^ to obtain a concentration of free Ca^2+^ that was close to the resting Ca^2+^ level in dark adapted *Drosophila* photoreceptors (Hardie, 1996). It is possible that this particular concentration was not suitable for cockroach transduction to function normally. In addition, the permeability profile of light-gated channels and the behavior of bumps in “0” [Ca^2+^] bath solution indicate that a significant fraction of the LIC could be mediated by an influx of Na^+^. It is consequently difficult to make solid conclusions about the sources of Ca^2+^ entering the cell. Nevertheless, an interesting finding was that in the brightest light level, the *Drosophila trp* phenotype could be reproduced with as well as without extracellular Ca^2+^ (Figure 9E). It is tempting to conclude that this behavior could result from the depletion of a relevant exciter (Hardie et al., 2001) or the prevention of rise in cytosolic Ca^2+^ through capacitive Ca^2+^ entry (Stieve and Benner, 1992; Ukhanov et al., 1995). However, a more thorough study is required to confirm this hypothesis.

The results obtained with bumps led us to believe that Ca^2+^ may not be such a predominant mediator of the LIC as it is in *Drosophila* photoreceptors (Figure 7; see also Henderson et al., 2000). The permeability profile of cockroach LIC revealed that this indeed seems to be the case as Ca^2+^ was only ~16 times more permeable than Na^+^. For comparison, in *Drosophila* photoreceptors, Ca^2+^ is roughly 39 times more permeable than Na^+^ (Reuss et al., 1997). Although the difference is not overtly dramatic it still could partly explain the lack of distinct effects with cockroach bumps while using different extracellular Ca^2+^ concentrations. The large size of Ca^2+^-free bumps also suggests that Na^+^ is responsible for a considerable fraction of the LIC. An intriguing possibility would be that cockroach photoreceptors also utilized TRP- and TRPL-type channels but with different proportions, favoring the amount of TRP less than in *Drosophila*. One of the remaining questions is also whether the Ca^2+^ influx via light-gated channels alone was enough to raise the intracellular Ca^2+^ levels sufficiently for proper regulation of transduction events.

### Conflict of interest statement

The authors declare that the research was conducted in the absence of any commercial or financial relationships that could be construed as a potential conflict of interest.

## References

[B1] Baumann O. Walz B. (1989). Calcium- and inositol polyphosphate-sensitivity of the calcium-sequestering endoplasmic reticulum in the photoreceptor cells of the honeybee drone. J. Comp. Physiol. A 165, 627–636 10.1007/BF00610994

[B2] Baylor D. A. Lamb T. D. Yau K.-W. (1979). Responses of retinal rods to single photons. J. Physiol. 288, 613–634 112243 PMC1281447

[B3] Burton B. G. (2006). Adaptation of single photon responses in photoreceptors of the housefly, *Musca domestica*: a novel spectral analysis. Vision Res. 46, 622–635 10.1016/j.visres.2005.09.020 16321420

[B4] Butler R. (1971). The identification and mapping of spectral cell types in the retina of *Periplaneta americana*. Z. Vergl. Physiol. 72, 67–80 10.1007/BF00299204

[B5] Butler R. (1973). The anatomy of the compound eye of *Periplaneta americana* L. J. Comp. Physiol. A 83, 223–262 10.1007/BF00693676

[B6] Chyb S. Raghu P. Hardie R. C. (1999). Polyunsaturated fatty acids activate the *Drosophila* light-sensitive channels TRP and TRPL. Nature 397, 255–259 10.1038/16703 9930700

[B7] Dorlöchter M. Stieve H. (1997). The Limulus ventral photoreceptor: light response and the role of calcium in a classic preparation. Prog. Neurobiol. 53, 451–515 10.1016/S0301-0082(97)00046-4 9421832

[B8] Fain G. Hardie R. C. Laughlin S. B. (2010). Phototransduction and the evolution of photoreceptors. Curr. Biol. 20, 114–124 10.1016/j.cub.2009.12.006 20144772 PMC2898276

[B9] Frolov R. V. Immonen E. V. Vähäsöyrinki M. Weckström M. (2012). Postembryonic developmental changes in photoreceptors of the stick insect Carausius morosus enhance the shift to an adult nocturnal life-style. J. Neurosci. 32, 16821–16831 10.1523/JNEUROSCI.2612-12.2012 23175835 PMC6621792

[B10] Frolov R. V. Immonen E. V. Weckström M. (2014). Performance of blue- and green-sensitive photoreceptors of the cricket *Gryllus bimaculatus*. J. Comp. Physiol. A 200, 209–219 10.1007/s00359-013-0879-6 24398538

[B11] Gu Y. Oberwinkler J. Postma M. Hardie R. C. (2005). Mechanisms of light adaptation in *Drosophila* photoreceptors. Curr. Biol. 15, 1228–1234 10.1016/j.cub.2005.05.058 16005297

[B12] Hardie R. C. (1991). Whole-cell recordings of the light induced current in dissociated *Drosophila* photoreceptors: evidence for feedback by calcium permeating the light-sensitive channels. Proc. R Soc. Lond. B 245, 203–210 10.1098/rspb.1991.0110

[B13] Hardie R. C. (1995a). Photolysis of caged Ca^2+^ facilitates and inactivates but does not directly excite light-sensitive channels in *Drosophila* photoreceptors. J. Neurosci. 15, 889–902 7529832 10.1523/JNEUROSCI.15-01-00889.1995 PMC6578327

[B14] Hardie R. C. (1995b). Effects of intracellular Ca2+ chelation on the light response in *Drosophila* photoreceptors. J. Comp. Physiol. A 177, 707–721 10.1007/BF00187630 8537938

[B15] Hardie R. C. (1996). INDO-1 measurements of absolute resting and light-induced Ca^2+^ concentration in *Drosophila* photoreceptors. J. Neurosci. 20, 1701–1709 8622123 10.1523/JNEUROSCI.16-09-02924.1996 PMC6579063

[B16] Hardie R. C. Franze K. (2012). Photomechanical responses in *Drosophila* photoreceptors. Science 338, 260–263 10.1126/science.1222376 23066080

[B17] Hardie R. C. Minke B. (1992). The *trp* gene is essential for a light-activated Ca2+ channel in Drosophila photoreceptors. Neuron 8, 643–651 10.1016/0896-6273(92)90086-S 1314617

[B18] Hardie R. C. Postma M. (2009). Phototransduction in microvillar photoreceptors of *Drosophila* and other invertebrates, in The Senses: A Comprehensive Reference: Vision I, eds Masland R. H. Albright T. D. (Oxford: Academic Press), 77–130

[B19] Hardie R. C. Raghu P. (2001). Visual transduction in *Drosophila*. Nature 413, 186–193 10.1038/35093002 11557987

[B20] Hardie R. C. Raghu P. Moore S. Juusola M. Baines R. A. Sweeney S. T. (2001). Calcium influx via TRP channels is required to maintain PIP_2_ levels in *Drosophila* photoreceptors. Neuron 30, 149–159 10.1016/S0896-6273(01)00269-0 11343651

[B21] Hardie R. C. Satoh A. K. Liu C. H. (2012). Regulation of arrestin translocation by Ca^2+^ and myosin III in *Drosophila* photoreceptors. J. Neurosci. 32, 9205–9216 10.1523/JNEUROSCI.0924-12.2012 22764229 PMC6622236

[B22] Hecht S. Shlaer S. Pirenne M. (1942). Energy quanta and vision. J. Gen. Physiol. 25, 819–840 10.1085/jgp.25.6.819 19873316 PMC2142545

[B23] Heimonen K. Immonen E. V. Frolov R. V. Salmela I. Juusola M. Vähäsöyrinki M. (2012). Signal coding in cockroach photoreceptors is tuned to dim environments. J. Neurophysiol. 108, 2641–2652 10.1152/jn.00588.2012 22933721

[B24] Heimonen K. Salmela I. Kontiokari P. Weckström M. (2006). Large functional variability in cockroach photoreceptors: optimization to low light levels. J. Neurosci. 26, 13454–13462 10.1523/JNEUROSCI.3767-06.2006 17192428 PMC6674726

[B25] Henderson S. R. Reuss H. Hardie R. C. (2000). Single photon responses in *Drosophila* photoreceptors and their regulation by Ca^2+^. J. Physiol. 524, 179–194 10.1111/j.1469-7793.2000.00179.x 10747191 PMC2269851

[B26] Hille B. (2001). Selective permeability: independence, in Ion Channels of Excitable Membranes (Sunderland, MA: Sinauer Associates, Inc.), 441–470

[B27] Howard J. (1983). Variations in the voltage response to single quanta of light in the photoreceptor of *Locusta migratoria*. Biophys. Struct. Mech. 9, 341–348 10.1007/BF00535669

[B28] Howard J. Dubs A. Payne R. (1984). The dynamics of phototransduction in insects: a comparative study. J. Comp. Physiol. A 154, 707–718 10.1007/BF01350224

[B29] Huang J. Liu C. H. Hughes S. A. Postma M. Schwiening C. J. Hardie R. C. (2010). Activation of TRP channels by protons and phosphoinositide depletion in *Drosophila* photoreceptors. Curr. Biol. 20, 189–197 10.1016/j.cub.2009.12.019 20116246

[B30] Keiper W. Schnakenberg J. Stieve H. (1984). Statistical analysis of quantum bump parameters in *Limulus* ventral photoreceptors. Z. Naturforsch. 39, 781–790 10.1007/BF01868736 2582123

[B31] Kelly K. M. Mote M. I. (1990). Avoidance of monochromatic light by the cockroach *Periplaneta americana*. J. Insect Physiol. 36, 287–291 10.1016/0022-1910(90)90113-T

[B32] Kirkwood A. Lisman J. E. (1994). Determinants of single photon response variability. J. Gen. Physiol. 103, 679–690 10.1085/jgp.103.4.679 8057084 PMC2216862

[B33] Lisman J. E. Richard E. A. Raghavachari S. Payne R. (2002). Simulatenous roles for Ca^2+^ in excitation and adaptation of *Limulus* ventral photoreceptors. Adv. Exp. Med. Biol. 514, 507–538 10.1007/978-1-4615-0121-3_31 12596942

[B34] Liu C. H. Satoh A. K. Postma M. Huang J. Ready D. F. Hardie R. C. (2008). Ca^2+^-dependent metarhodopsin inactivation mediated by calmodulin and NINAC myosin III. Neuron 59, 778–789 10.1016/j.neuron.2008.07.007 18786361 PMC2562427

[B35] Minke B. (1982). Light-induced reduction in excitation efficiency in the *trp* mutant of *Drosophila*. J. Gen. Physiol. 79, 361–385 10.1085/jgp.79.3.361 7077289 PMC2215757

[B36] Niemeyer B. A. Suzuki E. Scott K. Jalink K. Zuker C. S. (1996). The *Drosophila* light-activated conductance is composed of the two channel TRP and TRPL. Cell 85, 651–659 10.1016/S0092-8674(00)81232-5 8646774

[B37] Reuss H. Mojet M. H. Chyb S. Hardie R. C. (1997). *In vivo* analysis of the *Drosophila* light-sensitive channels, TRP and TRPL. Neuron 19, 1249–1259 10.1016/S0896-6273(00)80416-X 9427248

[B38] Running Deer J. L. Hurley J. B. Yarfitz S. L. (1995). G protein control of *Drosophila* photoreceptor phospholipase C. J. Biol. Chem. 270, 12623–12328 10.1074/jbc.270.21.12623 7759511

[B39] Salmela I. Immonen E. V. Frolov R. V. Krause S. Krause Y. Vähäsöyrinki M. (2012). Cellular element for seeing in the dark: voltage-dependent conductances in cockroach photoreceptors. BMC Neurosci. 13:93 10.1186/1471-2202-13-93 22867024 PMC3472236

[B40] Song Z. Postma M. Billings S. A. Coca D. Hardie R. C. Juusola M. (2012). Stochastic, adaptive sampling of information by microvilli in fly photoreceptors. Curr. Biol. 22, 1371–1380 10.1016/j.cub.2012.05.047 22704990 PMC3420010

[B41] Stieve H. Benner S. (1992). The light-induced rise in cytosolic calcium starts later than the receptor current of the Limulus ventral photoreceptor. Vision Res. 32, 403–416 10.1016/0042-6989(92)90232-8 1604827

[B42] Trujillo-Cenóz O. Melamed J. (1971). Spatial distribution of photoreceptor cells in the ommatidium of *Periplaneta americana*. J. Ultrastruct. Res. 34, 397–400 10.1016/S0022-5320(71)80080-1 5546718

[B43] Ukhanov K. Y. Flores T. M. Hsiao H. S. Mohapatra P. Pitts C. H. Payne R. (1995). Measurement of cytosolic Ca^2+^ concentration in *Limulus* ventral photoreceptors using fluerescent dyes. J. Gen. Physiol. 105, 95–116 10.1085/jgp.105.1.95 7730791 PMC2216928

[B44] Walz B. Baumann O. Zimmermann B. Ciriacy-Wantrup E. V. (1995). Caffeine- and ryanodine-sensitive Ca^2+^-induced Ca^2+^ release from the endoplasmic reticulum in honeybee photoreceptors. J. Gen. Physiol. 105, 537–567 10.1085/jgp.105.4.537 7608657 PMC2216935

[B45] Walz B. Zimmermann B. Seidl S. (1994). Intracellular Ca^2+^ concentration and latency of light-induced Ca^2+^ changes in photoreceptors of the honeybee drone. J. Comp. Physiol. A 174, 421–431 10.1007/BF00191708

[B46] Warrant E. J. Dacke M. (2011). Vision and visual navigation in nocturnal insects. Ann. Rev. Entomol. 56, 239–254 10.1146/annurev-ento-120709-144852 20822443

[B47] Wu C. F. Pak W. L. (1975). Quantal basis of photoreceptor spectral sensitivity of *Drosophila melanogaster*. J. Gen. Physiol. 66, 149–168 10.1085/jgp.66.2.149 809537 PMC2226201

[B48] Ye S. Leung V. Khan A. Baba Y. Comer C. (2003). The antennal system and cockroach evasive behavior. I. roles for visual and mechanosensory cues in the response. J. Comp. Physiol. A 189, 89–96 10.1007/s00359-002-0383-x 12607037

[B49] Yeandle S. Spiegler J. B. (1973). Light-evoked and spontaneous discrete events in the ventral nerve photoreceptor of *Limulus*. J. Gen. Physiol. 61, 552–571 10.1085/jgp.61.5.552 4705637 PMC2203478

[B50] Ziegler A. Walz B. (1990). Evidence for light-induced release of Ca^2+^ from intracellular stores in bee photoreceptors. Neurosci. Lett. 111, 87–91 10.1016/0304-3940(90)90349-E 2336197

